# Toward characterization of perceptual specialization for faces in Multiracial contexts

**DOI:** 10.3389/fpsyg.2024.1392042

**Published:** 2024-12-03

**Authors:** Charisse B. Pickron, Ethan Kutlu

**Affiliations:** ^1^Institute of Child Development, University of Minnesota Twin Cities, Minneapolis, MN, United States; ^2^Department of Linguistics, University of Iowa, Iowa City, IA, United States; ^3^Department of Psychological and Brain Sciences, University of Iowa, Iowa City, IA, United States

**Keywords:** perceptual specialization, Multiracial populations, face processing, infancy, developmental multilingualism

## Abstract

This conceptual analysis focuses on opportunities to advance research and current hypotheses of perceptual development by examining what is presently known and unknown about perceptual specialization in a Multiracial context during the first year of life. The impact of being raised in a Multiracial family or community is discussed to further characterize the development of perceptual expertise for faces and languages. Historical and present-day challenges faced by researchers in defining what race is, identifying Multiracial individuals or contexts, and how to study perceptual and cognitive processes in this population are discussed. We propose to leverage current data from developmental Multilingual populations as a guide for future research questions and hypotheses characterizing perceptual specialization based on face race for Multiracial/Multiethnic individuals and contexts. Variability of input and the pattern of specialization are two factors identified from the developmental Multilingual literature that are likely useful for studying Multiracial contexts and development. Several methodological considerations are proposed in hopes of facilitating research questions and practices that are reflective of and informed by the diversity of experiences and social complexities within Multiracial populations.

## Introduction

The lack of diversity in psychological research has been identified as one of the major concerns regarding reproducibility, replicability, and equity of scientific questions (Dunham and Olson, 2016; Gaither et al., 2015; Nishina and Witkow, 2020; Roberts et al., 2020; Singh et al., 2022). With the increased number of studies showing the lack of diversity, it becomes challenging to argue for the fairness of the current theories and tools scientists employ. The goal of this conceptual paper is to focus on what is missing in developmental psychology research in terms of understanding diversity (Singh et al., 2022; Tripp and Munson, 2023). Crucially, we focus on the intersection of race and language and argue that there are opportunities for furthering the current tools and theories toward inclusive practices and characterization of experiences in these domains. Particularly, the experience of Multiracial development which has received limited attention in the development of perceptual expertise, will be explored and discussed in this paper. Here, we aim to achieve this goal by focusing on what is done and what is missing in research to motivate future characterization of Multiracial/Multiethnic experiences shaping perceptual specialization across development. This is achieved through leveraging current work from simultaneous Multilingual perceptual expertise and show the intersection of these two by focusing on open questions that need to be addressed moving forward. The ultimate goal here is to present what is being missed in our current understanding of perceptual specialization when Multiracial experiences are not fully represented by our research questions or as participants in our studies across development.

## Situating Multiracial experiences in developmental psychology research

### What is race?

First, a grounding understanding that race is an ideology, constructed for economic and social power, often culturally engrained and psychologically salient (Salter et al., 2018; Smedley and Smedley, 2005). Race also plays a crucial role in forming group dynamics, and informs practices and policies while holding its hegemonic presence (Sekimoto, 2018). Race provides categories for general public as well as researchers to inform their decisions. In that regard, both scholars and general public essentialize these categories assuming that they provide robust [scientific] benchmarks (Hochman, 2021). But, even the labels used for racial categories are not representative of the population (Martinez, 2023). These labels are found to create ambiguous interpretation for scientists (Hochman, 2021).

Importantly, classifying race is also not an easy answer especially in Multiracial communities. This is especially evident when race categories intersect with ethnicity categories. Ethnicity is driven by shared customs, language(s), and may be racially homogenous or diverse (Smedley and Smedley, 2005). Through years of intersectional work, we know that there is erasure of distinctive experiences of marginalized communities. This is evident in cases of Multiracial and Multiethnic individuals where one dimension is ignored or receives more attention than the other one with the assumptions that these choices are power-neutral (Tripp and Munson, 2023; Umaña-Taylor et al., 2014).

### Who are Multiracial individuals?

The answer to this question greatly depends on from which perspective, culture, or country this topic is being considered (Song, 2021). The increasing body of literature highlights that racial diversity in one’s environment shapes social-group cognition, blurring or softening binary representations to more continuous category representations. One group which experiences this diversity is Multiracial individuals who have interracial experiences. In the U.S. alone Multiracial self-identified population increased by 276% in the past decade, with the Multiracial youth population increasing from 5.6% in 2010 to 15.1% in 2020 (Bureau, 2021). These individuals maintain more flexibility with one’s racial identity(s), and display less biased thinking (Gaither et al., 2015; Pauker et al., 2018; Pauker et al., 2016). However, perceptual development research still could obtain a richer characterization of variance within Multiracial individuals and experiences. Below, we situate some of the challenges faced thus far and then opportunities for shifting our research narratives to more inclusive and integrative ones.

## Historical shortcomings of research positioning Multiracial individuals

While there are various ways that this issue could be problematized, here, we start with three areas that need to be discussed further.

### The issue of baseline

For many decades, developmental psychology and cognitive psychology (along with many other social sciences) fields as well as publication practices (Roberts et al., 2020) pursued a more static approach in which Monoracially and Monolingually White and Western individuals with no disabilities are perceived as the baseline or modal exemplars of the whole population (Amir and McAuliffe, 2020; Henrich et al., 2010; McMurray et al., 2023; Muthukrishna et al., 2020; Nielsen et al., 2017). This practice is particularly visible through the types of research questions asked, stimuli used, demographic surveys, and participant recruitment. Despite cross-cultural evidence of similarly occurring perceptual narrowing during infancy, our working frameworks of when, why, and how such shifts appear in development are primarily understood from a Monoracial and Monolingual baseline. This results in many opportunities to think more inclusively and incorporate multi-input experiences as part of the baseline understanding of perceptual expertise.

### The issue of exclusion

Being Multiracial and/or Multilingual has historically been perceived as exclusion criteria. This practice leaves developmental science with a narrowed benchmark of what being a “representative” or generalizable participant is. This research practice is not exclusively driven by limited numbers of available participants. The exclusion of Multiracial and/or Multilingual individuals is supported by the fact that there still exist racialized ideologies in scientific places that suggest that being White and/or Monoracial/Monolingual is generalizable (Cheng et al., 2021; Nielsen et al., 2017) with minimal flexibility of category boundaries. The critical emphasis we raise below, is to focus on opportunities to shift *away* from excluding those with multi-category experiences to *including* them into developmental science.

### The issue of reporting

One of the most used data collection methods in psychological sciences is the use of demographic surveys. These surveys are used to report participant demographics for three reasons: (i) publication, (ii) reporting for agencies, and (iii) statistical analysis control variables. While questioning the validity of demographic surveys is a whole different venue to be explored, the lack of specificity in traditional surveys makes it challenging to fully characterize Multiracial and Multilingual developmental experiences (Surrain and Luk, 2019). However, in recent years some researchers are making the shift to include more open-ended participant-identity focused questionnaires offering richer representations of individual variability of experience (e.g., Atkin et al., 2022; Byers-Heinlein et al., 2020; Ellis et al., 2017; Roberts et al., 2020).

## Brief literature review of face processing within developmental Multiracial experiences

Racial categories are heavily investigated in face perception research. The capacity to discriminate among exemplars within a particular category represents specialization with the most common input (Fort et al., 2024; Lewkowicz, 2014; Quinn et al., 2019). Infants raised primarily in a racially homogenous and/or Monolingual home develop a perceptual system fine-tuned to matching face race and language (for reviews, see Maurer and Werker, 2014; Pascalis et al., 2014; Scott et al., 2007). This process is termed perceptual narrowing and is believed to be a domain general process reflecting perceptual specialization. An interaction of top-down and bottom-up attentional, perceptual and social motivational systems are believed to drive this specialization (Hadley et al., 2014; Markant and Scott, 2018; Scherf and Scott, 2012). Perceptual specialization is evidenced through declines in discrimination capabilities and neural responsivity to unfamiliar category exemplars as well as maintenance and/or improvements for highly familiar categories (Aslin and Pisoni, 1980; Scott et al., 2007; Werker, 2024). Infants experience shifts in visual differentiation capabilities for multiple face categories including race (Kelly et al., 2007), cross-species (Simpson et al., 2011; for a review see Scott and Fava, 2013), and face gender (Quinn et al., 2010; Rennels et al., 2017).

Monoracial infants, those raised by caregivers in racially homogenous households and environments, show more robust differentiating, detecting, attending, and responding to faces (and related facial cues) of a familiar race relative to unfamiliar races. For example, 3-month-old Chinese infants differentiate among both Chinese and Black-African faces, however by 9 months, they only reliably differentiate among Chinese faces (Kelly et al., 2007). A similar pattern is found for U.S. White infants, with a decline in differentiating among Black faces as well as face-voice matching between 6 and 9 months of age (Vogel et al., 2012). Despite these robust and replicated findings, it is not an indication of a critical period, with the face processing system consolidating at 9 months post birth. Researchers show that this pattern of perceptual narrowing is malleable based on naturally occurring living circumstances (Bar-Haim et al., 2006; Hwang et al., 2020; Rennels et al., 2017) or through experimental manipulation (Anzures et al., 2012; Markant et al., 2016; Minar and Lewkowicz, 2018; Pascalis et al., 2005; Pickron et al., 2018; Quinn et al., 2020; Scott and Monesson, 2010; Scott and Monesson, 2009). But does perceptual narrowing and the development of perceptual specialization for processing “own−/familiar-” vs. “other−/unfamiliar-” race faces vary as a function of being raised in Multiracial environments?

### Multiracial societies

A racially diverse out-of-home context can provide increased opportunities for a multiplicity of input across race and language. Several studies have broadly examined the way racially diverse societies shape developmental perceptual specialization for familiar-versus unfamiliar-race faces (Bar-Haim et al., 2006; Ellis et al., 2017; Hwang et al., 2020; Tham et al., 2019). For example, U.S. 8-month-old White infants with out-of-home exposure to Individuals of Color displayed distinctive eye movement strategies for unfamiliar-race faces not observed in age-matched White infants without such experiences (Ellis et al., 2017). Greater neural responses thought to reflect top-down attentional control and approach systems toward unfamiliar-race faces was found in U.S. White infants from neighborhoods with larger racial diversity (Hwang et al., 2020). These findings may indicate racial diversity, with even minimal contact, improves perceptual engagement with unfamiliar-race individuals. Furthermore, out-of-home racial diversity may improve Monoracial individuals’ face discrimination of out-group members. For example, 3- to 4-month-old and 8- to 9-month-old Chinese infants living in the racially diverse and integrated city of Kuala Lumpur, Malaysia were tested for face differentiation among familiar-race Chinese faces, more-experienced other-race Malay faces and less-experienced other-race White faces (Tham et al., 2019). Younger infants displayed face discrimination capabilities only for familiar-race female Chinese faces. These findings contradict the straw-man conclusion that 3-month-old infants’ face discrimination capabilities are perceptually most flexible and un-biased (e.g., Kelly et al., 2007, 2009). However, this bias is consistent with 3-month-old Monoracial infants living in a Multiracial U.S. city (Gaither et al., 2012). The 8-to 9-month-old Chinese infants displayed face discrimination capabilities for own-race Chinese and the more-experienced other-race Malay female faces, but not for less-experienced White faces (Tham et al., 2019). These findings indicate a broadening or facilitation pattern of perceptual discrimination may result from out-of-home Multiracial experiences. This broadening pattern is consistent with infants’ face-gender discrimination capabilities (Rennels et al., 2017; Righi et al., 2014). When receiving multiple inputs, infants’ face perception system may first tune to the exemplar most salient and then develop expertise for the next most experienced input. This possible pattern is opposite to homogenous Monoracial experiences that begin broad and then quickly narrow.

### Multiracial individuals

Despite the dramatic population increase of Multiracial youth, those raised with caregivers not of the same race, there are very few reports on perceptual specialization for infants raised within a racially mixed insular family. Gaither et al. (2012) tested United States 3-month-old Asian-White Multiracial infants, who all had Asian mothers and White fathers, on a face discrimination task. Asian-White Multiracial infants did display greater proportion of looking to the novel relative to familiarized White face, however, did not show this discrimination for familiar-race, Asian, face comparisons. These findings leave us with many questions about the trajectory of perceptual specialization throughout the first years of life and across other Multiracial familial contexts.

### Beyond infancy

Despite possible broadening or other patterns of perceptual specialization throughout infancy, narrowing for familiar-race(s) faces may eventually develop in childhood. For example, a small sample of Black-White US Biracial participants ranging from 5 years to 23 years of age displayed no differences between face memory scores relative to Monoracial participants (Goodman et al., 2007). Starting at 8 years of age participants displayed the classic, familiar-race bias, showing better memory for familiar-race White as well as Black faces relative to unfamiliar-race faces. These findings suggest that for these Black-White Biracial children their face perception expertise formed for the two race groups they are most exposed to. More data is needed to understand if a pattern of narrowing to more than one race group is consistently found across all Multiracial children and/or contexts.

Societal or individual racial diversity alone may not fully characterize developmental perceptual expertise in childhood. Children develop their own sense of identity along with learning about social categories such as race and gender through explicit labels and implicit behavioral messages (Bigler et al., 2006; Quintana, 1998). For example, 3- to-5-year old child with more experience to racial-labels also have neural responses indicative of implicit category formation based on face race (Timeo et al., 2019). The specificity of familiar-group biases may become even more specialized for some children raised in diverse contexts, especially those who are in marginalized groups. An example of the way ethnicity also plays a role in perceptual expertise in childhood is found in 5- to 16-year-old Asian children of different ethnic backgrounds (e.g., Chinese, Filipino, and Korean). These children failed to display a familiar-race face bias when tested with faces rated as racially Asian, but critically not of the same Asian *ethnicity* as the children who were tested (Gross, 2013). In contrast, White children succeeded in displaying a familiar-race bias for racially White faces of varying ethnicity. Gross (2013) speculated that because many neighborhoods in Southern California are segregated by race *and* ethnicity, many of the Asian children in the study had more own-ethnicity exposure whereas, White children likely had own-race exposure without ethnic diversity. This is evidence for when ethnic diversity supersedes race in salience for in-group face specialization, despite being situated in a Multiracial society (See Boxes 1, 2, for more). Work by Gross (2013) highlights that experience with environmental racial and ethnic diversity can be both interconnected and distinct in the influence of developmental perceptual specialization. More work is needed to evaluate the way these different categories of faces may influence face perception biases which begin during infancy. Likely, social systems of hierarchy and experiences of marginalization play a macrolevel role for determining when ethnicity or race are more salient for an infant’s or child’s experiences with face diversity and development of face processing biases.

Overall, the reviewed findings indicate there may be several varying pathways to developing specialization for faces of familiar-race group(s) compared to unfamiliar groups. Throughout the next sections brief summaries of the reported similarities and differences between Monolingual and simultaneous Multilingual speech perception are provided as a guide to shaping future face perception research with Multiracial populations. Then the overlap between developmental Multilingual and Multiracial individuals regarding perceptual specialization is considered and highlights two overarching themes that shape current and future research related to these populations. There are also several open questions proposed (see Box 1) that will push our research toward a more inclusive and robust characterization of perceptual specialization.

BOX 1 **Open questions for face perception research.**

These questions are aimed to extend exploration of race-related face perception within Multiracial contexts.

- How do Multiracial and Multiethnic experiences compare in developing perceptual expertise for in-group faces? Researchers will need to examine when and how facial experiences grouped by race compare to being grouped by ethnicity influence perceptual expertise? Secondly, researchers will need to characterize these differences across development. Should we expect to see ethnic-group based perceptual biases as early as race-group biases or are such sensitivities interconnected to later developing socio-cognitive biases. It is likely that Multiethnic experiences impacting in-group face processing biases will relate to prioritization levels of racial categorization within a given context along with an individuals’ group membership.
- Are there substantive differences in developing familiar-race specialization based on out-of-home environments compared to in-home family experience? Variance in the source of multilingual context appears to drive individual-level variability for Multilingual perceptual capabilities (Birdsong, 2018 for review). What qualitative differences between out-of-home racially diverse environments relative to multiracial in-home environments are expected to impact perceptual specialization?
- Links between socio-political systems, out-of-home Multiracial environments & face-race specialization? It is encouraged that further exploration of links between system-level practices and individual-level face-race specialization. Is out-of-home Multiracial context and subsequent unfamiliar-race perceptual sensitivities likely to differ based on the socio-political systems of group-level stratification? For example, should we predict similar findings from Malaysia (Tham et al., 2019) for perceptual specialization from infants in South Africa or Puerto Rico? The potential link between social systems and out-of-home Multiracial exposure and developing face-race specialization is particularly salient in countries rich in history of cross-group conflict, marginalization, and (increasing) Multiracial populations.
- How do top-down factors of racial identity and ethnic-racial socialization influence perceptual specialization for familiar-and unfamiliar-race faces? When primed with a single racial identity, Multiracial adults shift perceptual categorization of another’s race toward the primed racial identity (Gaither et al., 2016). More work is needed to understand this face perception–racial identity link and its development. Caregivers’ family language policies shapes the way Multilingual children are exposed to their multiple languages (Quay and Montanari, 2016). Ethnic-racial socialization (ERS, Hughes et al., 2006) may be a parallel process by which children learn about race relations. It is unclear the way caregiver ERS may influence perceptual processes in early development. ERS scales for Multiracial populations (Atkin et al., 2022) may offer opportunities to examine links between parenting practice and perceptual specialization.

## Discussion

Faces and voices independently and collectively provide a wealth of cues resulting in predictions about someone’s familiarity, personal identity, emotion, age, gender identity, and racial category membership (Campanella and Belin, 2007; May et al., 2019; Pascalis et al., 2014; Perrachione et al., 2010; Tripp and Munson, 2021). It is hypothesized that there are averaged face and averaged voice constructs that are used to evaluate perceptual and social likeness when experiencing novel exemplars (e.g., Latinus and Belin, 2011; Valentine, 1991). These averaged familiar spaces develop from the extensive experience and the statistical regularity of specific types of inputs infants have during the first year of life (Kuhl et al., 1992). Speech and face perception are interconnected through shared processing systems (Yovel and Belin, 2013).^1^ Developmental evidence of this shared system is demonstrated through the overlapping, though not perfectly correlated (Xiao et al., 2018), timing of perceptual narrowing for familiar-language and familiar-race faces (Krasotkina et al., 2021; Krasotkina et al., 2018; Maurer and Werker, 2014).

Face-language multisensory perceptual narrowing further exemplifies this link when investigating perceptual expertise during the first year of life (Lewkowicz, 2014). For example, 6-month-old Spanish-learning infants visually differentiated between the audiovisual presentation of one woman producing an English /ba/ versus another woman producing a /va/ (Pons et al., 2009). In contrast, 11-month-old Spanish-learning infants failed to differentiate between the two talking women. The failure to differentiate by 11 months reflects multisensory perceptual narrowing and is believed to occur because Spanish does not differentiate between /b/ and /v/ sounds. These findings indicate that perceptual narrowing for familiar language occurs across both auditory and visual modalities. Infants’ visual fixations within a face shift from the eyes to the mouth and back to the eyes between 4 and 12 months of age (Lewkowicz and Hansen-Tift, 2012). These findings are interpreted as a reflection of infants’ becoming experts at word production between 8 and 12 months of life and no longer needing the intersensory redundancy of a moving face. Interestingly, this visual shift in fixation strategies back to the eyes at 12 months are not observed when infants are presented with an audiovisual talking face speaking an unfamiliar language (Lewkowicz and Hansen-Tift, 2012). The perceptual decline in sensitivity for unfamiliar-race faces may even be improved for 9-month-old infants when paired with an unfamiliar-language, suggesting perceptual and early categorical associations based on face race and language familiarity (Clerc et al., 2021). However, the effect of face-language pairing for learning familiar race faces appears to be more complex with varying effects based on modality (motion vs. static) of face images (Hillairet de Boisferon et al., 2021).

One of the areas where face-language pairing becomes relevant is when listeners are asked to listen to various accents. Developmental research shows that children (5–6 year old) prefer friends who sound like them and, if they do not hear a voice, they prefer same-race children as friends. The same age group was also shown to prefer individuals who have similar accents regardless of race. More studies on adults’ perception of language and race suggest that listeners make quick judgments about a person’s speech intelligibility when they see a face on a computer screen (Kutlu, 2020; Kutlu et al., 2022b). These judgments are shown to be mediated by racial bias, lack of linguistic and racial diversity, and Multilingual/Multiracial experiences (Kutlu et al., 2022c). One framework, particularly in the area of language sciences, is called raciolinguistic framework which focuses on the ways in which race and language influence the perception of a person (Rosa and Flores, 2017).

### Brief literature review of developmental multilingualism research

The majority of the world’s population is Multilingual or Multidialectal (Grosjean, 1989; Kirk, 2023) and quite possibly Multiracial and/or Multiethnic (Aspinall, 2018). However, this is not well reflected in our research practices, much of it is driven by differences in how these social identity categories are defined as well as recognized across the world. These also lead to the (mis)conceptualization that Multilingual or Multidialectal individuals are two Monolinguals or Monodialectals in one mind (Grosjean, 1989). Thus, many theoretical frameworks are situated to understanding individuals of these populations’ experiences, perceptual, and cognitive development from a deviation from the “mono” context.

As speech is perceived as continuous sound, so too may the social construct of racial categories. The perception, representation, and navigation of racial continuum may be particularly salient for Multiracial individuals or those raised in racially diverse and integrated contexts (Nishina and Witkow, 2020). Research indicates similarities and differences between Multilingual and Monolingual infants when it comes to familiar language perceptual specialization. Many of the differences seem to fall within two overarching themes of perceptual specialization (A) variability of input and (B) the pattern of specialization. It is beyond the scope of this paper to offer an in-depth review of developmental Multilingualism literature, however in the following paragraphs a variety of reported patterns and exemplary evidence are highlighted to lay a foundation for the type of questions and content we are missing for familiar-race face specialization for Multiracial experiences. While we overview these studies, we also need to highlight the limitations of the Multilingualism research overviewed below. The majority of the developmental Multilingualism research has had emphasis on simultaneous Multilingualism from specific regions of the world (Rocha-Hidalgo and Barr, 2022; Singh et al., 2022) and without the careful consideration of many issues that minoritized Multiracial and Multiethnic Multilinguals face in everyday life. Furthermore, Multilinguals’ experiences of minoritization through their language practices has a direct effect on racialization of these individuals. Therefore, the intersection of race and language becomes the crucial point in understanding Multiracial and Multilingual individuals’ cognitive processes (see Kutlu et al., 2022a, Kutlu et al., 2022b for examples from adult populations).

### Variability of input

Timing, duration, context, and modality of input varies both within and between individuals and societies. These variations of input, particularly perceptual, are all critically influential to developmental neuroplasticity that defines much of the first years of life (for review, see Oakes, 2017). Variability of input has cascading and multidirectional influence on perceptual specialization output during infancy, which in turn shifts future input experiences for the developing child (Oakes, 2017). We see this input variability is both salient and a shared characteristic for those raised in Multilingual/dialectic and Multiracial/ethnic families as well as societies.

Often all Multilinguals are considered to have less input due to the input being split in different languages. Whereas the overall amount of time hearing speech sounds may not vary between Multi-and Monolingual infants (Kalashnikova and Carreiras, 2022), Multilingual infants are hearing more variation within this time. Time spent with a single home language may be lower because of Multilingual infants negotiating between languages (Byers-Heinlein and Fennell, 2014; Hoehle et al., 2020; Orena et al., 2020) and home languages being marginalized in many contexts (Rocha-Hidalgo and Barr, 2022). The source of linguistic exposure and amount of time exposed to each language are two key qualities for describing Multilingualism (Rocha-Hidalgo and Barr, 2022; Sebastian-Galles and Santolin, 2020). There are similar complexities and some nuances when considering the variability of input for Multiracial experiences.

From simultaneous Multilingual literature, authors report a correlation between reported minimum time spent with multiple languages and significant perceptual discrimination findings. For example, infants with more unbalanced dual-language exposure, seem to show greater differences in perceptual discrimination relative to Monolinguals compared to those infants with more balanced dual-language exposure (Bijeljac-Babic et al., 2012; Sebastián-Gallés and Bosch, 2002). This is also reported for Multidialectal infants, with greater variability of accent input broadens older infants’ capability of deciphering within-category linguistic variance (Potter and Saffran, 2017).^2^ Additionally, bilingual infants who hear more mixing of two languages from their mothers appear to be better at segmenting across their dominant and non-dominant languages compared to those infants with low experiences of language mixing (Orena and Polka, 2019).

However, the majority of these studies did not consider the effects of societal barriers on these infants’ environment. For instance, do these infants have access to multiple languages in and outside of the home? Are there any societal pressure on parents regarding maintaining one language over the other one? Crucially, there is now advancements in Multilingualism research that suggest that when we use continuous measures to capture fundamental processes in spoken language such as phoneme categorization, it becomes evident that what was interpreted as deficiency or lack of discrimination ability in Multilinguals become a gradient categorization pattern (Kutlu et al., 2022a). We argue that developmental psychology research should adopt these approaches instead of pushing for an understanding that language and faces are non-variant. When we start with the assumption that input is varied, then we observe that Multilingualism experience offers insights to understand how we cognitively adapt to the variation that surround us.

#### Links to out-of-home Multiracial context

Regional, cultural, and socio-political practices are macro-system level factors shaping the likelihood of being raised in racially homogenous relative to diverse and integrated environment. For example, in the US structural racism strategically makes for racially homogenous environments to be far more common and often desired than racially integrated spaces (Adelman and Gocker, 2007). When racial integration does occur at community or neighborhood level there is evidence that perceptual capabilities change (Bar-Haim et al., 2006; Ellis et al., 2017; Hwang et al., 2020; Kutlu et al., 2022c; Kutlu et al., 2022b; Tham et al., 2019; Tiv et al., 2022), yet understanding the link between variation in this type of community exposure and individual-level perceptual outcome needs further investigation.

Overall, the contextual differences for linguistic exposure may drive individual variability even within developmental Multilingual populations. From these findings, it may be expected that infants raised in Multiracial environments where racial boundaries are perceived or socially defined more clearly (due to racial segregation practices) may show perceptual sensitivity for the multiple groups they are in contact with. This race-salient context may result in perceptual specialization like Monoracially raised infants, but the specialization is for more than one face race group. For infants raised in contexts where racial category boundaries are not as salient or there is more socio-politically endorsed racial integration, perceptual discrimination capabilities may be even more flexible or less discrete along racial category constructs (see Box 1 for additional considerations linking face perception to socio-political practices).

#### Links to Multiracial family

Multilingual individuals’ perceptual sensitivities range from being balanced between languages to having a dominant language (Birdsong, 2018). Such individual-level variability results from linguistic variance of input within and outside of the family (Birdsong, 2018; Quay and Montanari, 2016) as well as societal demands on Multilingual individuals and the minoritization of their language use (Flores and Rosa, 2015; Rosa, 2016). The link between language perception dominance and input variability for Multilingual infants may transfer well for considering variability in face-race perceptual specialization within Multiracial families. There are of course infinite ways a Multiracial person presents, socializes, and is raised within Multiracial families. Here are four cases exemplifying in-home Multiracial family experiences that may result in varied face-race perceptual input: (1) An infant with two racially different Monoracial caregivers, (2) A family with racially different Monoracial caregivers, with older Multiracial children and a younger infant. (3) All family members are multiracial, and (4) A single Monoracial caregiver with a Multiracial [or transracially adopted De Heering et al. (2010)] infant. Racial categorization is largely driven by visually observable physical features and verbal labels. In these hypothetical family examples, the amount of perceivable categorical distinctions further contributes to the complexities in hypothesizing and generalizing patterns of familiar-race face specialization. Each of these Multiracial family contexts may offer a Multiracial infant different amount (and likely quality) of perceptual racial diversity that in turn will shape the timing and pattern of familiar-race perceptual specialization. To add onto this complexity, these infants might also receive language input that may be stigmatized at the societal level. These scenarios may result in varying degrees of perceptual dominance for more than one familiar-race category. This complexity also raises critical need to re-evaluate the assumptions and decisions made by researchers developing new studies with Multiracial populations (see Box 2).

BOX 2 **Methodological considerations prior to collecting data.**

Researchers are encouraged to evaluate the following questions before collecting data on group-based familiar face perceptual processes, particularly when studying Multiracial populations and contexts (see Rocha-Hidalgo and Barr, 2022 for Multilingual considerations).

- What does Multiracial mean? It is inefficient to argue for a static definition of dynamic and socially defined populations. However, is there a minimum amount of time within racially diverse context to label someone as having Multiracial experience and to expect shifts in perceptual specialization? Infants are identified as Multilingual if 20–25% of linguistic exposure is from multiple languages (Rocha-Hidalgo and Barr, 2022). Is time (e.g., 25%) a justified metric for researchers to use to define Multiracial experiences? If not, what other qualities are needed for categorizing one’s experiences as Multiracial?
- Whose face is in my “own”-race? Which races fall within a Multiracial infants’ familiar-race face category? Two key components will need to be considered. First, is there a single, most familiar, race category for every infant regardless of racial diversity within the infants’ environment? Secondly, who else besides primary caregivers should be included in considering an infants’ racial in-group?
- How do we decide which races? The answer depends on who and how researchers ask the question. The *who* refers to if researchers decide to use participant data or researchers’ own *a priori* empirical or methodological needs are prioritized. The *how* refers to whether questions are open response or categorical selections of racial groups and cumulative time. Will each race category be equally weighted within the familiar-race category, or should a gradient approach be taken? Using a gradient approach, researchers then need to operationalize the levels of familiarity. Racial familiarity may depend on quantity of time as well as group size and/or number of racial groups represented.
- What about the face stimuli? Inclusion of Multiracial participants requires reflection on the type of stimuli used in our research. We sit at a crossroads of relying on stimuli reinforcing essentialism of discrete racial categories while simultaneously including participants who blur category boundaries. How do we move forward with increasingly complex racial representation and our research materials?
- Should we move toward questions of familiar ethnicity instead of race? More data is needed to characterize the way face perception among ethnic groups may be just as sensitive to experience as race group familiarity. Researchers will need to consider both racial and ethnicities represented within their sample and face stimuli.

### Timing and pattern of specialization

The second overarching theme is the way infants from racially diverse contexts may achieve specialized perceptual capabilities for familiar-race relative to unfamiliar-race faces. Evidence from the handful of previously reviewed investigations examining face-race perception offer initial evidence for both similarities and differences between Multiracial individuals and racially homogenous Monoracial individuals (Gaither et al., 2012; Iankilevitch et al., 2020; Tham et al., 2019; Tham et al., 2017; Woo et al., 2020). Two starting points are proposed to tackle this large theme: Timeline for specialization and what (perceptual) “narrowing” may look like.

#### Timeline

By 12 months, both Monolingual and Multilingual infants display evidence of perceptual learning through maintaining and improving discrimination for frequently heard phonemes, stress patterns, and rhythms in their familiar-language(s; for reviews, see Hoehle et al., 2020; Singh, 2021; but also see Apfelbaum et al., 2022; McMurray, 2022 for reviews of task-related variance). Behavioral and electrophysiological data indicate that although by 4 months Monolingual infants are readily differentiating familiar language from unfamiliar languages regardless of similarity levels, Multilingual infants may be doing so in a different manner. Specifically, instead of displaying faster latencies to familiar-language sounds as Monolingual infants, some Multilingual infants are showing faster latency responses to unfamiliar-languages (Bosch and Sebastián-Gallés, 1997; Nacar Garcia et al., 2018). In some cases, simultaneous Multilingual infants display prolonged onset timing for robustly biased perceptual discrimination within their familiar-languages (for review, see Singh, 2021). For example, before 12 months Bilingual infants, particularly those with more equal distribution of dual-language input, display neural activity indicative of being less mature or less fine-tuned to native speech sounds relative to Monolingual infants (Ferjan Ramírez et al., 2017; Garcia-Sierra et al., 2016; Garcia-Sierra et al., 2011). Another reported difference is the U-shaped curve, describing a developmental shift from discriminating familiar speech sounds prior to 6 months, an apparent absence of this from 6 to 8 months and then a reemergence around 10 to 12 months (Bosch, 2018; Bosch and Sebastián-Gallés, 2003a, 2003b; Byers-Heinlein and Fennell, 2014; Sebastián-Gallés and Bosch, 2009). However, contrasting evidence shows that when languages are highly distinct there is no evidence of the U-shaped curve (Sundara et al., 2008; Sundara and Scutellaro, 2011). Bosch (2018) proposes language proximity is a likely driver for mixed results of the U-shape pattern as well as other prolonged timing for linguistic sensitivities reflective of specialization. To be clear, findings of differences in this context do not reflect deficits, instead the overall conclusion is that Multilingual infants may display an extended timeline in the onset of narrowing to familiar-language and speech content relative to unfamiliar-language. This is potentially driven by contextual needs that these infants need to adopt to. Alternatively, the tasks used in many of these studies which are geared toward ignoring variation (i.e., forced choice tasks) are not great in capturing variability observed in Multilingual language development (Apfelbaum et al., 2022; Kutlu et al., 2022a). It is currently unclear whether we should predict similar specialization onset timing variability for Multiracial infants.

It appears that the closer in overlap between learned languages are, the more prolonged the perceptual specialization timeline may be for some Multilingual infants. The potential for a prolonged window of specialization may be particularly relevant for those who are in family or community contexts with high amount of Multiracial representation. Thinking about race along a continuum, it could be argued that Multiracial type contexts hold opportunity for higher amounts of ambiguity and less clear boundaries for developmental perceptual systems to generate averaged spaces defining familiar-race and unfamiliar-race categories. With more racial ambiguity and increased contact of multiple groups it is possible that like Multilingual infants learning highly proximal languages, infants of Multiracial contexts may display perceptual specialization intermittently or not as robustly until into the second year of life. This would suggest that in contexts of ambiguity, a face-race perceptual system is not as constrained or motivated to become fine-tuned for a particular group. In contrast for Multiracial infants or racially diverse contexts where racial categories are perceptually and/or socially salient, the onset of perceptual specialization may occur more quickly or along the same timeline as Monoracial infants. The construct of race is increased in psychological salience both through interpersonal interactions (e.g., label use) and society or cultural practices (e.g., systemic racism; for reviews see, Bigler et al., 2006; Salter et al., 2018; Waxman, 2021). In both cases infants and young children learn the practices and develop associations and/or categories of race.

#### Patterns of narrowing?

**Figure 1:**
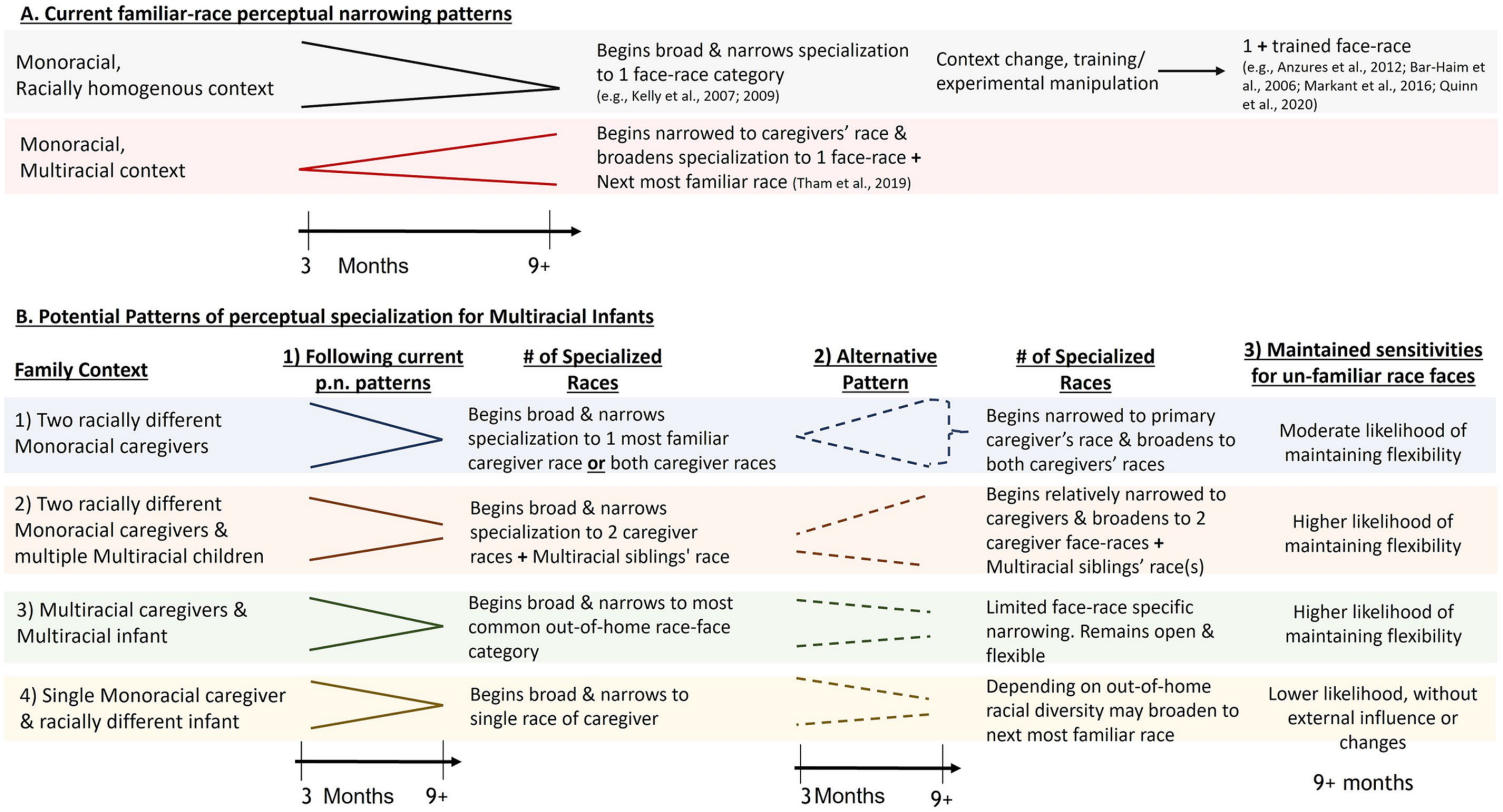
Current and Predicted Patterns of Face Perception Expertise. **(A)** Depicts a summary of findings related to perceptual narrowing testing Monoracial infants. The direction of the vector represents narrowing or broadening patterns of perceptual capability. **(B)** Offers 4 hypothetical Multiracial family contexts and 3 possible perceptual specialization patterns. Pattern 1, with the solid lines are predictions following the current perceptual narrowing literature. Pattern 2, dashed lines, depicts alternative possibilities including opposite trends from Pattern 1. Pattern 3 offers possibly likelihood of infants maintaining perceptual sensitivities for un-familiar race faces after 9 months of age.

Perceptual expertise for familiar-race faces is typically described as a narrowing pattern, however for Multiracial individuals and contexts, specialization may follow several different types of patterns. The predicted type of pattern will likely depend on the previously described factors of input variability and when such racial diversity is occurring along with the macro level societal structure. Based on the limited current data conducted either with Multiracial individuals or those raised in a Multiracial context, perceptual differentiation capabilities may start relatively narrowed to the most familiar race and then increase in capability to the next most familiar face-race. However, more research is needed with more racially representative samples and age groups to fully grasp the potential nuances for the developmental pattern of perceptual specialization. There are at least three other plausible directions that familiar-race face perception specialization could take. First, Multiracial infants develop perceptual expertise for faces of a singular most-experienced race group like Monoracial infants. Second, Multiracial infants develop perceptual expertise for more than one familiar-race category, but still decline in capabilities for other unfamiliar-race faces. Third, their perceptual sensitivity remains open or does not close at all between 3 and 9 months of age. Figure 1 depicts these possible specialization patterns and which type of Multiracial family context they may be more likely to occur in (i.e., the previously described four hypothetical Multiracial families). These alternative hypotheses are consistent with the proposed “perceptual wedge” symbol, that the experience of learning more than one (language) generates a wedge that keeps the perceptual discrimination door open (Petitto et al., 2012).

The idea of maintained perceptual flexibility for unfamiliar categories has been examined for simultaneous Multilingual infants (Burnham et al., 2018; Petitto et al., 2012; Sebastián-Gallés et al., 2012). For example, 18-to 20-month-old English/Mandarin Bilingual infants displayed learning of an unfamiliar South African language that age-matched monolingual infants did not display (Singh, 2018). Perceptual and learning advantages driven by the experienced linguistic variability seems to extend to accent perception (Potter and Saffran, 2017) and unfamiliar face-language mapping (Fecher and Johnson, 2019). One study examined whether Multilingual infants’ perceptual flexibility extended across to the face perception domain. Specifically, 11-month-old Chinese-English Bilingual infants displayed maintained perceptual discrimination capabilities for unfamiliar, non-native Hindi phonemes, but did not display face discrimination capabilities for unfamiliar-Black faces compared to familiar-race Chinese faces (Singh et al., 2017). Their poorer performance for unfamiliar-race faces matched those of the monolingual-Chinese infants, suggesting that perceptual flexibility found within the Bilingual infants may be limited to the linguistic domain. There is not enough data to make strong predictions for Multiracial/Multiethnic infants’ maintenance of perceptual sensitivities. Individual differences in the amount of time spent within a Multiracial context as well as in what capacity, will likely influence infants’ perceptual openness and flexibility for unfamiliar race faces (see Figure 1B, 3rd Pattern).

## Future directions and concluding remarks

Multiracial/Multiethnic and Multilingual/Multidialectal contexts do not solely occur independently of one another. Limited research has already shown that Multilingual infants and children have lower social bias for unfamiliar race individuals (Singh et al., 2020; Singh et al., 2019), though familiar-race perceptual narrowing still occurs (Singh et al., 2017). When infants are raised with simultaneous linguistic and racial diversity should we expect “super” perceivers with perceptual plasticity above those who are either Multilingual or Multiracial? Alternatively, might these dual multi-input experiences shed light on attentional and perceptual thresholds of plasticity for the developing brain? This type of perceptual domain cross-over offers the field even more opportunities to characterize the dynamic relation between perception, experience, and later-occurring socio-cognitive development.

Here, we emphasize the need for more Multiracial research through the comparative lens of using Multilingual studies as an example. However, both of these identities should not be exclusively studied (as in the case of Monoracial and Monolingual identities). We argue that more inclusive studies with Multiethnic and Multidialectal contexts will also move us away from the issues of reproducibility by allowing us to capitalize on equity and justice in research.

There is a clear overlap in the development of perceptual expertise for our familiar language and race groups. Leveraging the simultaneous Multilingual literature to propose new avenues of inquiry to advance characterization of perceptual processes developing within Multiracial contexts. Distinctions between homogenous singular contexts and how a multiplicity type input does, and likely will, alter the way perceptual specialization takes place throughout the first year of life have been made. There are two overarching factors critical for shaping future research: Variability of input and pattern of specialization. With these themes we are better able to consider the way increased visual racial variability results in narrowing and/or broadening one’s perceptual capabilities throughout development. Ultimately, our research questions should go beyond the hegemonic understanding of the society which argues for Monolingual and Monoracial individuals neither of which captures the variability observed in the population. Taking our research beyond these singular categories may further support alleviation of our field’s replication challenges through capturing truer or more generalizable representation of experiences.

Studies evaluating infants from Multiracial contexts will elevate the inclusivity and robust characterization of the relation between early experience and perceptual specialization. Initial studies indicate that infants raised within a Multiracial home or racially integrated urban society display face processing patterns that differ from Monoracial-racially homogenous infants (e.g., Gaither et al., 2012; Tham et al., 2019). These initial studies offer a critical launching point for researchers to fill gaps regarding the populations represented and the age of infants studied. Thus, we are left with an exciting and wide-open landscape to think deeply and question our current perspectives of face processing and developmental specialization. Studying face processing expertise within Multiracial populations will also contribute to bridging the gap in understanding of the way early perceptual processes lay foundation for later developing socio-cognitive processes. It is likely that perceptual specialization is inevitable, and the varied pathways by which this process occurs, and the full diversity of environmental inputs that shape it, warrant further interrogation. We show here that our scientific pursuits have been systematically scratching at the same side of this case and diverse perspectives are needed in order to better inform our theories.

## Footnotes

^1^We recognize that these linked processes and experiences are likely distinct for those who have varying degrees of hearing and/or vision impairments (e.g., Grady et al., 2014; Guerreiro et al., 2016).

^2^We note that multidialectal and accent variability are reported both as interchangeable and completely distinct concepts in the linguistic literature. It is beyond the scope of the current paper to devel into this specificity. In the current paper we report on literature that has used these terms interchangeably.
